# The Interaction of Large Amplitude Internal Seiches with a Shallow Sloping Lakebed: Observations of Benthic Turbulence in Lake Simcoe, Ontario, Canada

**DOI:** 10.1371/journal.pone.0057444

**Published:** 2013-03-05

**Authors:** Remo Cossu, Mathew G. Wells

**Affiliations:** 1 Department of Physical & Environmental Sciences, Earth Sciences Centre, University of Toronto, Toronto, Ontario, Canada; 2 Department of Physical & Environmental Sciences, University of Toronto, Toronto, Ontario, Canada; Plymouth University, United Kingdom

## Abstract

Observations of the interactions of large amplitude internal seiches with the sloping boundary of Lake Simcoe, Canada show a pronounced asymmetry between up- and downwelling. Data were obtained during a 42-day period in late summer with an ADCP and an array of four thermistor chains located in a 5 km line at the depths where the thermocline intersects the shallow slope of the lakebed. The thermocline is located at depths of 12–14 m during the strongly stratified period of late summer. During periods of strong westerly winds the thermocline is deflected as much as 8 m vertically and interacts directly with the lakebed at depth between 14–18 m. When the thermocline was rising at the boundary, the stratification resembles a turbulent bore that propagates up the sloping lakebed with a speed of 0.05–0.15 m s^−1^ and a Froude number close to unity. There were strong temperature overturns associated with the abrupt changes in temperature across the bore. Based on the size of overturns in the near bed stratification, we show that the inferred turbulent diffusivity varies by up to two orders of magnitude between up- and downwellings. When the thermocline was rising, estimates of turbulent diffusivity were high with *K_Z_* ∼10^−4^ m^2^s^−1^, whereas during downwelling events the near-bed stratification was greatly increased and the turbulence was reduced. This asymmetry is consistent with previous field observations and underlines the importance of shear-induced convection in benthic bottom boundary layers of stratified lakes.

## Introduction

Turbulent mixing at the sloping boundaries of a lake is a key process for transporting nutrients into the water column where they can become available for the growth of plankton. In stratified lakes the thermocline is always in motion due internal seiches caused by wind forcing. The movement of internal seiches near the sloping boundary can energize benthic boundary layers. Hence the level of turbulence strongly increases along the boundaries [1], and the vertical turbulent diffusivities (*K_z_*) in benthic bottom boundary layers (BBLs) are usually orders of magnitude larger than in the quiescent interior of stratified lakes (e.g. [2], [3], [4]). Consequently, BBL mixing is of critical importance to the water quality of a lake due to its significance for nutrient fluxes from lake sediments into the water column [1] or the transport of substances such as oxygen, microorganisms and pollutants [5]. However, direct observations of the BBL turbulence are limited, and it remains unclear how intermittent the turbulence is and how the magnitude of the turbulent diffusivity depends upon meteorological forcing upon a lake.

The wind driven tilting of the thermocline in a stratified lake can energize the BBL turbulence by several distinct mechanisms, with breaking internal waves and shear induced convection having received much recent attention. Non-linear internal waves (NLIW) can form in a lake due to steepening of an internal seiche [6] assuming that the seiche can last for several oscillation periods before being dissipated by friction [7]. These NLIW are hypothesized to then shoal and break in near-shore regions [3], [8], [9], [10], [11]. NLIW are frequently observed in the ocean, where there is a long time for non-linear processes to steepen waves. Many observations reveal that NLIW break and form strongly energized BBL on the coastal margin (e.g., [12], [13]). However while NLIW are sometimes directly observed in large lakes [14], [15], [16], there are very few direct observations that conclusively show them breaking at a lake boundary at the depth of the thermocline.

A second process that can lead to energized mixing of a BBL by an internal seiche is called shear-induced convection. This process involves the differential advection of stratified waters near a sloping boundary during up- and downwelling events due to internal seiching [4], [17], [18], [19]. During periods of upslope flow, a velocity gradient forms near the boundary, so that away from the boundary layer water is moving faster. If there is a background stratification, the upslope flow advects dense water over lighter water, leading to density inversions. This process is termed as shear-induced convection [17]. During downslope flow, lighter fluid is preferentially advected over dense fluid leading to an enhanced stratification [19] which can restabilize the BBL [17], [19], [20]. Thus shear-induced convection can cause a strong asymmetry in the level of BBL turbulence and stratification between up- and downwelling phases of the internal seiche.

The presence and role of shear-induced mixing in BBLs has been closely studied in the oceanographic context (e.g., [12]). For instance, shear-induced convection is similar to tidal straining in estuaries, but in lakes it is initiated by internal seiches instead of tides [21], [22]. Observations of shear-driven convection in lakes stem from two different single point observations in Lake Alpnach [17] and Lake Constance [4]. The results from these point measurements are consistent with analysis of numerical simulations of shear-driven convection [18], [19], [23]. Nonetheless, to date there are only a few field observations in lakes leaving it unclear as to the relative importance of shear-induced convection versus breaking of NLIW in energizing benthic BBLs.

In this paper we report observations of thermal variability and associated shear-induced mixing in the BBL on the shallow slope of a large lake. Data were recorded at four thermistor chains over a 42 day period in late summer during strong stratification of the water column. We relate observations of large thermocline movements associated with the internal seiche to the occurrence of strong benthic temperature fluctuations. We discuss the asymmetry between the up- and downwelling phases of the internal seiche, where both the thermal stratification and inferred turbulent diffusivities change as the thermocline moves up and down along the shallow slope. In particular, we show strong differences in both the benthic stratification and the duration of upwelling compared to downwelling. Increased benthic turbulence is shown to be quite intermittent, and we finish with a discussion of how the infrequent large upwelling events lead to episodic, turbulent events in the BBL of Lake Simcoe.

## Field Observations Description

### Field Site Description

**Figure 1:**
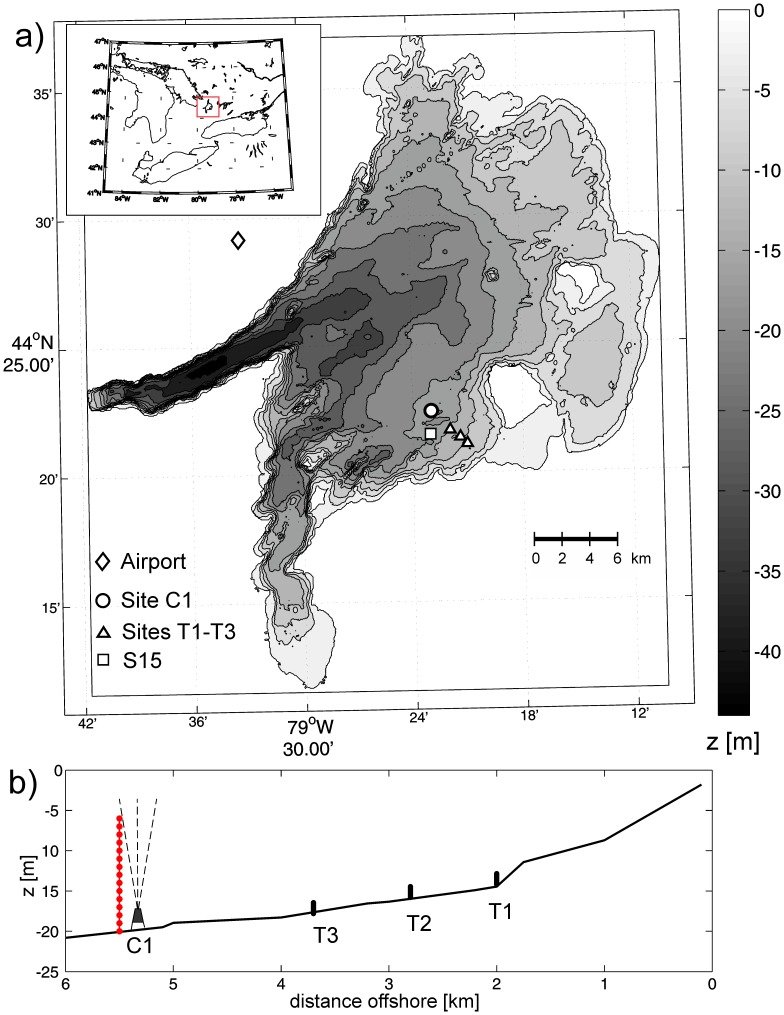
Field experiment location and bathymetry of Lake Simcoe, Ontario (Canada). a) Bathymetry of Lake Simcoe. Four thermistor chains were arranged in a cross-shelf transect, to depths between 20 m and 12 m (contours shown in m). An acoustic Doppler current profiler (ADCP) was located next to the 20 m mooring. Also shown is the location of Barrie Airport (weather station) and the long term MOE monitoring site S15. b) Alignment of four thermistor chains (C1, T1–T3) and the ADCP in the cross-shelf transect of the southern shore of Lake Simcoe.

The study was conducted in Lake Simcoe (44°25′N, 79°30′W), a freshwater, dimictic lake located in southern Ontario, Canada. Lake Simcoe is the sixth largest inland lake of Ontario and of great value for recreational uses such as boating and fishing, and is an important local drinking water supply. However, since the 1970s water quality problems have been documented and attributed to the increased agricultural, urban and industrial use of Lake Simcoe and its watershed [24], [25]. The lake is composed of one main basin with two side arms; in the west is Kempenfelt Bay and in the southwest is Cook’s Bay (Figure 1). The total surface area is 722 km^2^ and the volume is 11.6 km^3^. The main basin is roughly circular with a gently sloping shallow eastern half, and steep slopes on the western side. The field site is located on the south-eastern shore of the main basin where there is a gentle slope of approximately 1.5 m per km. The lake has a maximum depth of 41 m, with a mean depth of 20 m and a maximum effective fetch of 30 km (Figure 1). Lake Simcoe is oligotrophic with a mean thermocline depth of 10 m, where typical summer time surface temperatures reach 20–22°C in the epilimnion and 8–10°C in the hypolimnion [25].

No permits are required to moor temperature loggers and ADCPs in Lake Simcoe. There was no sampling of any fish, hence no permits were required under the “Fish and Wildlife Conservation Act” of the Ontario Ministry of Natural Resources, which is the only relevant legislation. This work was done in full consultation of the Lake Simcoe Regional Conservation Authority and the Ontario Ministry of the Environment, whose boat and crew were used to deploy the instruments.

## Materials and Methods

An array of instruments was deployed offshore from day of year (DOY) 215 to 258 (3^rd^ of August until 15^th^ of September 2011). The 4 moorings were assembled in a line from shallow (44°21.12′N 79°21.03′W) to deeper areas (44°22.4′N 79°22.98′W) depicted in Figure 1. The deepest mooring was a vertical thermistor chain (C1) deployed at a depth of approximately 20 m, 5 km from the southern shoreline at 44°22.44′N 79°23.08′W. This is 1 km north of the long term Ontario Ministry of the Environment monitoring site S15 (44°21.86′N 79°23.27′W). The long thermistor chain at C1 consisted of 16 individual temperature loggers (TR-1060, RBR), which were located between 5 and 20 m in depth, with a vertical spacing of 1 m. The loggers had an accuracy of 0.002°C and the sampling interval was 4 s. In addition, three vertical benthic thermistor chains were deployed near the lake bottom in a line between the long thermistor chain and the shore at depths of 17.8 (T3), 15.8 (T2) and 14.2 m (T1). The location and relative distance to shore is depicted in Figure 1b. Each benthic thermistor chain consisted of 10 individual temperature loggers (Seabird SBE 56), which were located between 0.05 and 1.40 m above the lakebed, with a vertical spacing of 0.15 m. The accuracy of these thermistors is 0.002°C, and the sampling interval was set to 2 s. The internal clocks of all loggers were synchronized at the beginning of the sampling period. All data presented here start at the DOY 216 (0∶00) approximately 8–12 h after the instruments were deployed in the water and finish at the DOY 257 of 2011. Velocity data were collected using an RDI 600 kHz, 4 beam acoustic Doppler current profiler (ADCP) moored on the bottom near to C1 at a depth of 20 m (Figure 1). The ADCP recorded velocities of the 4 beams (Mode 1) every 30 seconds in bins of 100 cm starting from 2.3 m above the lake bottom. 55 pings were averaged per sample and the uncertainty for 30 s intervals was 0.0095 m s^−1^. Wind speed and direction, air temperature and relative humidity were obtained from the hourly records of the Environmental Canada meteorological station located at the Barrie airport (44°29′′N 79°33′′W ) on the western shore of Lake Simcoe.

### Parameters

The density of the water was computed from the measured temperature *T* using the freshwater equation of state [26]. The position *h* of the seasonal thermocline was determined by calculating the first moment of the density gradient every 4 s
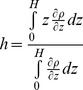
(1)with *z* being the vertical coordinate and *ρ* the density of water, to identify the location of maximum density gradient [27].

The dimensionless Lake number *L_N_* was first formulated by [28] and is used to give an indication of the strength of the wind compared to stratification. We used the numerical code Lake Analyzer [29] to obtain time series data for *L_N_* from the observed stratification and wind record. The Lake number is defined by [29] as
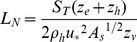
(2)where *A_s_* represents the surface area of the lake, *z_e_* and *z_h_* are the depth to the top and the bottom of the metalimnion, *z_v_* is the depth of the center of volume of the lake and *ρ_h_* is the density in the hypolimnion. The water friction velocity u* is estimated from the measured wind speed as 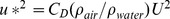 where *C_D_* is a drag coefficient (*C_D_* = 10^−3^ for winds <5 m s^−1^ and *C_D_* = 1.5×10^−3^ for winds >5 m s^−1^ [29]), *U* the observed wind speed, and 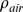 the density of air. *S_T_* represents the Schmidt stability and is defined as
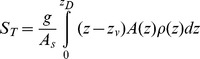
(3)where *A(z)* is the area of the lake and *ρ(z)* is the density as a function of *z* respectively. *z_D_* is the maximum depth of the lake. In (2) and (3) we take 12 hour running averages of both u* and density. In small lakes *L_N_* determines the magnitude of the thermocline tilt due to the wind forcing [30] and is also indirectly related to the turbulence in the water column [1], [28], [31]. When *L_N_* is less than order 1, surface upwelling of deep water will occur at the upwind end of the lake [28] and turbulence is predicted to be enhanced within the lake. This was shown in recent studies where enhanced mixing in the BBL was observed to occur when *L_N_* <2, in the 20 km wide Mono Lake [1] and in the 5 km long Lake Opeongo [30]. In larger lakes, such as Lake Michigan, Coriolis forces will also be important, so that the magnitude and location of upwelling events is also influenced by Ekman transport [32]. The upwelling will be mainly on the shore that is to the left of the winds direction, and downwelling will occur to the right. As Lake Simcoe is 30 km wide, we may expect Coriolis forces to be somewhat important in determining the initial location of surface upwelling. However, we will only use *L_N_* as a rough indication of the strength of the wind forcing to the thermocline tilt. As our experiment was restricted to a small area in the southern part of the lake we neglect a full analysis on the influence of the Earth’s rotation in this study.

The intensity of turbulence in a stratified water column can be characterized by the occurrence and magnitude of temperature inversions in the water column [15], [33], [34] which represent gravitationally unstable conditions [17]. In this study temperature inversions were defined to occur when the temperature difference between two vertically separated thermistors at heights *z_1_* and *z_2_* exceeds *T*(*z_1_*) –*T*(*z_2_*) ≤ −4×10^−3^°C where the height of the thermistors are such that *z_1_*> *z_2_*. This threshold is twice as high as the accuracy of the temperature loggers, and also represents the average of the range of values used in other studies to identify density inversions, e.g., −5×10^−4^°C in [35] or −0.01°C in [15]. In addition, it is similar to the threshold of −3×10^−3^°C which was used in [4] to estimating the occurrence of 1m overturns and to which we compare our results to.

The length-scale of these temperature inversions is used to estimate the vertical turbulent eddy diffusivity *K_z_*. An estimate of the rate of dissipation of turbulent kinetic energy (*ε*) can be made from the Thorpe length scale *L_T_* using
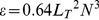
(4)where we have used the relationship that the Thorpe length scale *L_T_* is related to the energy containing Ozimodov scale, as 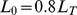 [35], hence the factor of 0.64. The Thorpe length scale is obtained by reordering the raw temperature profiles into a stable monotonic profile without inversions and the length scale of displacements *d* is calculated as the vertical distance that an element must move to create a stable monotonic temperature profile [33], [36]. For each turbulent patch the Thorpe scale is defined as 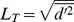. The buoyancy frequency *N* is computed from the reordered stable temperature profile and is calculated as 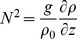 with *g* being the gravitational constant, *ρ* is the density of water and *z* is the depth measured from the surface. The vertical turbulent diffusivity *K_z_* is then calculated as
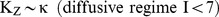
(5a)

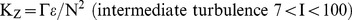
(5b)

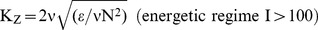
(5c)


where *Γ* is the mixing efficiency, 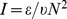 is the turbulence activity parameter which defines different turbulent mixing regimes [37], ν is the dynamic viscosity with ν = 1.004×10^−6^ m^2^s^−1^ and the molecular diffusivity of heat is *κ = *1.4×10^−7^ m^2^s^−1^ near the boundary where *T*∼ 20°C. The mixing efficiency *Γ* is equal to the maximum value of 0.2 [37], [38] for the intermediate turbulent regime.

## Results

### Meteorology

**Figure 2:**
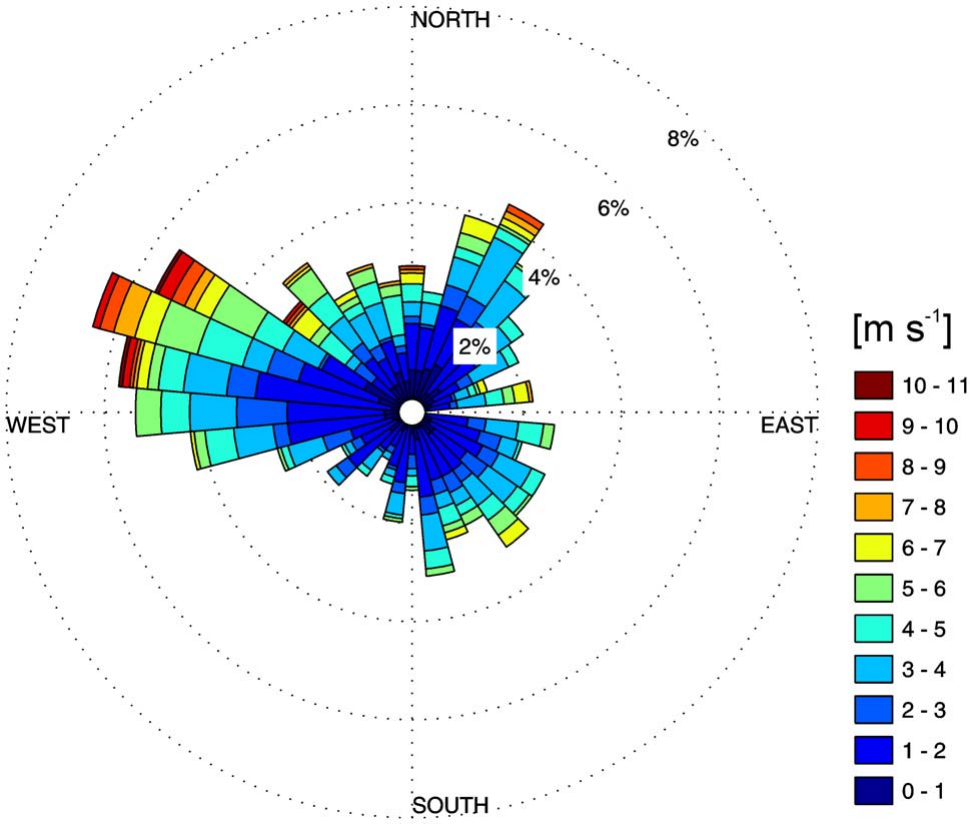
Wind rose for the Barrie Airport Weather Station for August and September 2011, i.e. DOY 216–257. Direction indicated is that from which wind is blowing. Concentric rings (2, 4, 6 and 8) give percentage of time wind is from that particular direction; colors within a wedge represent the relative proportion of time the winds from that direction are of a certain magnitude.

Analysis of long-term wind data from Environment Canada indicates that the prevailing winds are westerly for most of the year, particularly so for the summer. This is shown in the wind rose in Figure 2 for the period of the field observations. The strongest wind events with speeds exceeding 10 m s^−1^ on two occasions came from the west/northwest and were almost perpendicular to the south shore and therefore to the location of the field site in Lake Simcoe. The mean wind speed during the field observations was 2.75 m s^−1^ which is slightly less than the long-term averages of 3–3.5 m s^−1^ during this time of the year. Wind-generated waves were not recorded in 2011 due to a problem with the Environment Canada wave-rider buoy. However, wave parameters analyzed in Lake Simcoe for similar conditions in 2010 show that the waves are strongly fetch-limited in this lake and have significant wave heights less than half a meter. As the wave period is also less than 3 seconds, their wavelength is usually much smaller than the depth of the thermocline, and they are expected to play a relatively minor role in the deepening the thermocline, or leading to turbulence near the bed in the pelagic zone. Thus waves are not considered in this analysis.

### Stratification at Pelagic and Benthic Thermistor Chains

**Figure 3:**
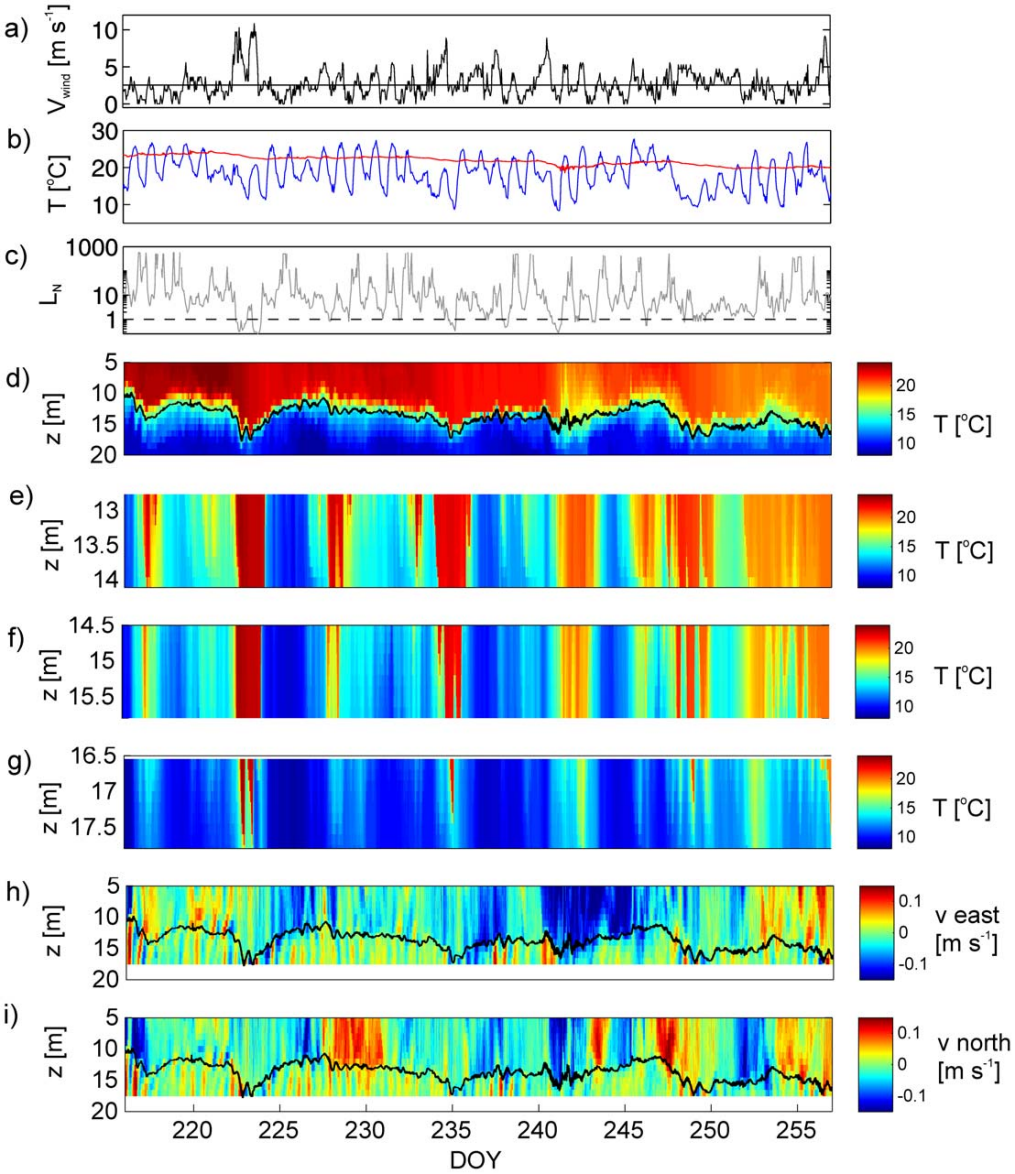
Observations of stratification, wind and currents in Lake Simcoe. a) Recorded wind speed at Barrie Airport. b) Comparison of air temperature (blue line) and water temperature (red line) at the top thermistor chain 5 m below the surface. c) Estimated Lake number calculated with a 12 hour moving average of wind speed and stratification, the dashed line represents a value of 1. d) The thermal stratification is shown with color contours, with the position of the thermocline at the C1 site marked in black. e-g) Temperature stratification observed at T1 to T3 during up- and downwelling of the thermocline. h) ADCP velocities east. i) ADCP velocities north.

The main observations are summarized in Figure 3 and show how movements of the thermocline associated with internal seiches are driven by wind forcing. In Figure 3a, the mean wind speed is represented by a horizontal line, and on several occasions the wind briefly exceeded 10 m s^−1^ (Figure 3a). The air temperature exhibits a diurnal cycle of cooling and mixing (Figure 3b) and the water temperature records from the top thermistor (5 m below the surface) indicate values constantly above 24°C with slightly higher values at the beginning and decreasing values towards the end of the period. The Lake number drops to values significantly below 1 for at least 5 times, when wind speeds exceed 8 m s^−1^ (Figure 3c). Records from the long pelagic thermistor chain reveal that there is considerable variation in the depth of the thermocline (black line) during the observations (Figure 3d), with the location of the thermocline varying between depths of 10 m and 17.8 m. Typical vertical movements of the thermocline over a day are less than 2 m but these large movements of the thermocline indicate large internal waves (seiches). The largest internal seiches coincided with strong winds and usually occur after *L_N_* has dropped below 1. Several strong thermocline excursions down to depths near the lakebed can be identified, such as the significant events between DOY 222–225 and DOY 242–244 (Figure 3d). At the beginning of the observations Lake Simcoe was already stratified. During the experiment the average depth of the thermocline increased from 10 m to 15 m. After DOY 235 the temperature difference across the thermocline starts to weaken due to cooling from the now cooler air temperatures.

Considerable thermal variability is also very evident at the 1.4 m high benthic thermistor chains located at the lakebed (Figure 3e to 3g). Changes in temperatures at T1 to T3 coincide with movements of the thermocline associated with large amplitude seiches that were recorded at the deeper thermistor chain C1. When there is a large amplitude seiche, the thermocline directly intersects the lakebed. The temperature at the lakebed can then vary between 8°C and 24°C as the thermocline moves up and down. The thermal variability is greatest at depths close to the mean depth of the thermocline, so that the shallowest site T1 at the depth of 14.2 m experiences the most frequent and longest periods of temperature fluctuations. Generally, cooling of water at the benthic sites is attributed to upwelling of cold water whilst warm periods represent times when warm water is transported to deeper areas due to downwelling of the thermocline.

### Observation of Currents

The ADCP measurements of the along-shore and cross-shelf velocity data are illustrated in Figure 3h and 3i. The data are taken very close to the C1 thermistor chain, and reveal a strong baroclinicity in the form of a distinct two-layer structure separated by the thermocline (black line). The strong periodicity of velocities in the bottom layer can be linked to the movements of the thermocline associated with internal seiches. Strong and prolonged velocities of order 0.1 m s^−1^ are seen in the upper water column, during and after strong winds, e.g. DOY 223–230, 235–238 and 240–245. Maximum velocities near the bottom reach 0.15 m s^−1^ and were predominantly recorded after the upwelling phase of the internal seiche, such as in the periods at DOY 223, 235 and 243.

### Temperature Inversions

**Figure 4:**
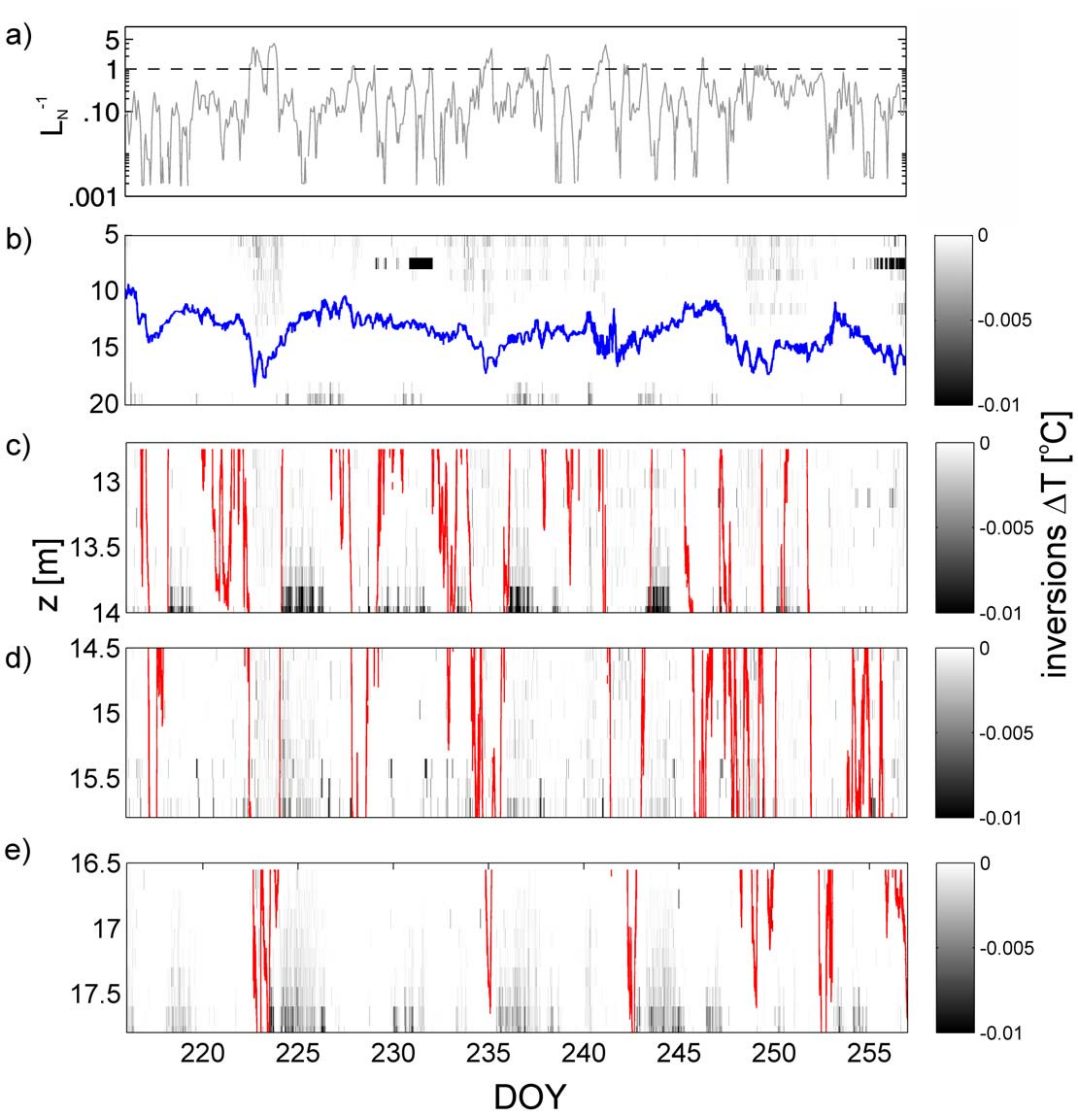
Observed temperature inversions in the water column. a) Inverse of the estimated Lake number *L_N_*. b) Inversions at C1 mooring. Note that the blue line represents the position of the thermocline at the thermistor chain. c) Inversions at T1. d) Inversions at T2. e) Inversions at T3. The red line reflects the local 16°C isotherm (local thermocline) for each benthic thermistor chain.

When the movements of the thermocline were most active, temperature inversions where seen near the bed at all four moorings (Figure 4b to 4d). This occurred especially after strong wind events when *L_N_* is small (Figure 4a) and the thermocline has been strongly deflected so that seiches had their strongest amplitudes. Inversions occur in the upper water column in Figure 4b primarily due to wind-induced shear at the surface or convection due to cooling. In contrast, near the bottom periodic temperature inversions only occur during the upwelling phases of the internal seiche. These unstable conditions can extend over the full 1.4 m of the benthic moorings, although they usually have a scale of several tens of cm (Figure 4c–4e).

**Figure 5:**
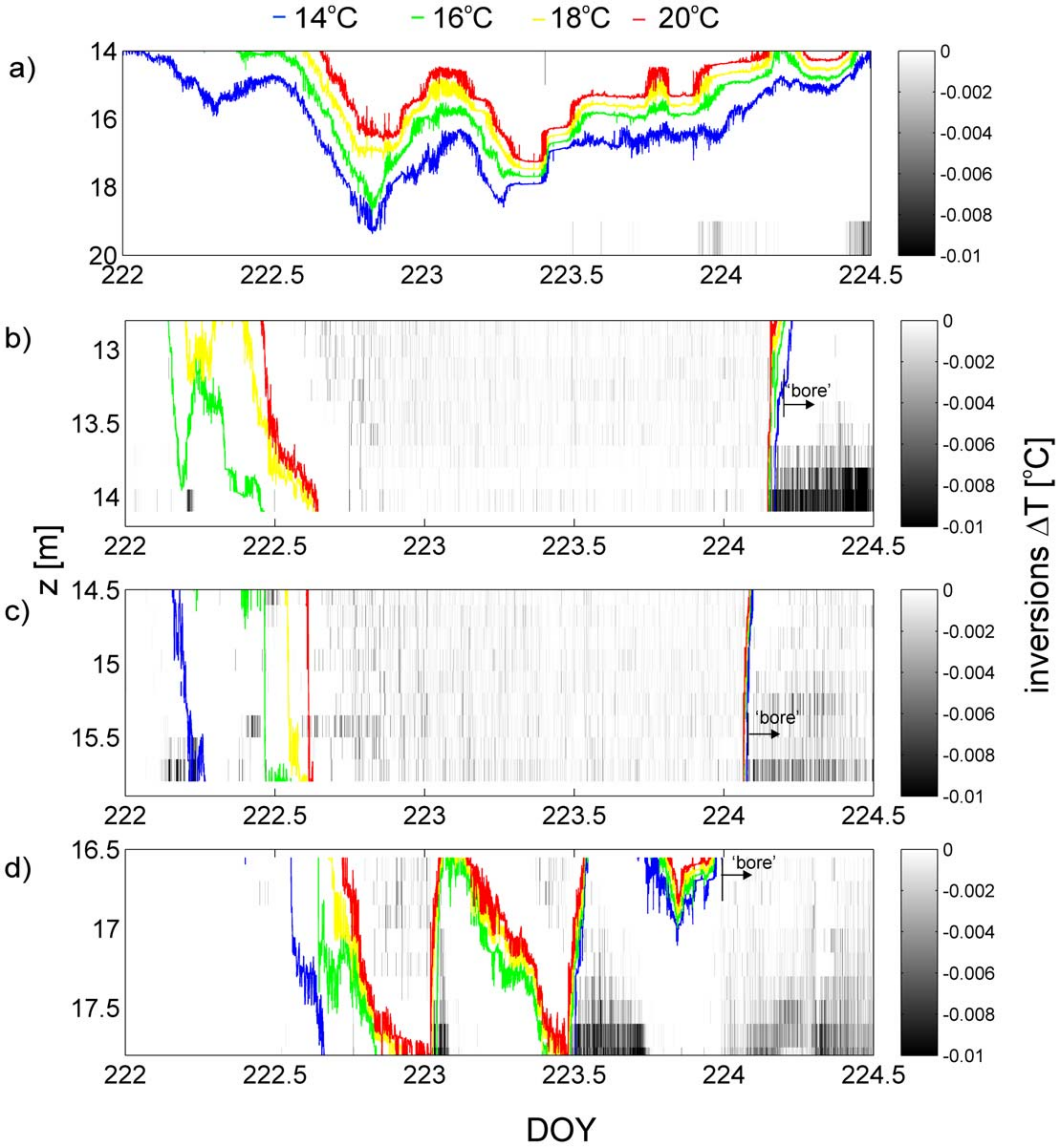
Relation between local isotherm movements and temperature inversions. Data are shown at the four thermistor chains during and after a strong storm event between DOY 222 and 225 at a) C1. b) T1. c) T2. d) T3. The blue, green, yellow and red lines denote the position of the local 14°C, 16°C, 18°C and 20°C isotherm at each site. The position of the cold water front (bore) is based upon the steep temperature gradients in Figure 3e–3g which show the upslope flow of a cold bore into shallower waters. The arrival of the bore is represented by rapid cooling (steep gradient of the isotherms) followed by the onset of temperature inversions.

A detailed description of the relation between temperature inversions and thermal variability after a strong wind event is presented in Figure 5 which shows a full cycle of an internal seiche, e.g. an up- and downwelling event. The inversions measured at C1 are shown in Figure 5a and the benthic thermistor chains T1 to T3 are presented in Figure 5b–d. It is evident that during the upwelling phase of the internal seiche, the local isotherms at the lake boundary are not at the same location as might be expected from extrapolation of the offshore thermocline. Rather there is a pronounced delay, so that for instance the local 16°C isotherm is rising at the boundary several hours after it has risen offshore. The upwards passage of the 16°C isotherm at the boundary is almost immediately followed by the onset of strong and prolonged temperature instabilities associated with shear-induced convection at the lakebed (Figure 5b–d). In contrast, during the downwelling phase of the internal seiche there are relatively stable conditions with almost no temperature inversions at the lakebed.

## Discussion

### Interaction of Seiches with the Lake Bed

There is a strong asymmetry in the benthic temperature variability between the up- and downwelling phases of the internal seiche. This is particularly evident in the time series of benthic temperatures in Figure 5 where downwelling and warming between the 14°C and 20°C takes 12 hours (shown by large temporal spacing between the isotherms) whereas the upslope flow of cold water leads to rapid cooling near the bottom (the isotherms are very concentrated temporally). In contrast there is no pronounced difference in the behavior between the up- and downwelling of the thermocline at the offshore site C1. The upwelling of cold water leads to a destabilized BBL, as indicated by the temperature inversions, whereas the downwelling of warm water actually results in an increased stratification. Such asymmetries in up- and downwelling are consistent with the description of shear induced convection [17], [19], [20], and are concurrent with observations in the ocean on the continental slope, which have revealed asymmetries in properties of BBL during upwelling and downwelling of the oceanic thermocline [39], [40].

The very rapid decrease in the water temperatures measured within the bottom 1.4 m at all benthic thermistor chains (Figure 5b–d) as the thermocline rises offshore, indicates that the movement of thermocline at the boundary is much like that of a non-linear internal bore. The movement of the internal bore can be tracked in Figure 5, starting when the 16°C isotherm (red line) is elevated above 16.5 m at T3. Approximately 2 hours after that the cold water bore can be seen at T2 and subsequently later at T1. This internal-bore-like structure is accompanied by overturning temperature inversions which have been attributed to an increase in velocity away from the boundary so that colder water is continually being advected over warmer water [14], [19].

**Figure 6:**
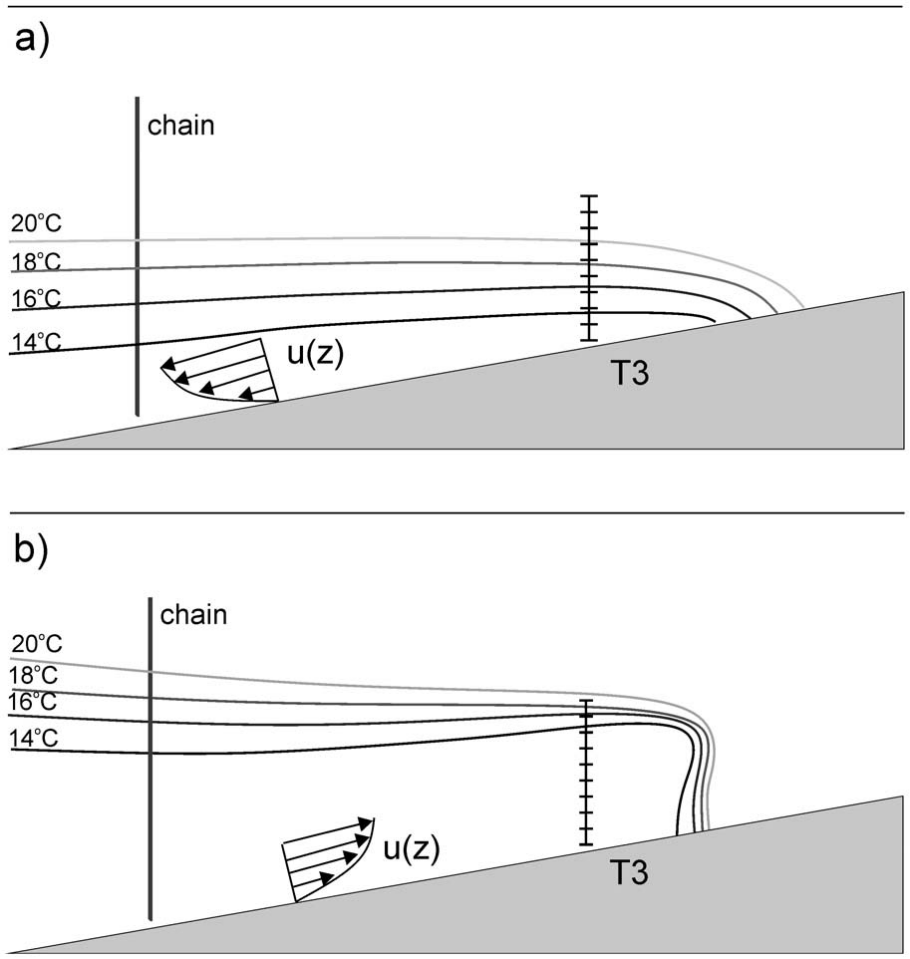
A schematic diagram describing the asymmetry in stratification between up and downwelling events. The position of isotherms is shown at both the deep mooring and the benthic mooring. a) During downwelling events there is a stable stratification in the water column. b) During upwelling events the isotherms resemble a ‘bore-like’ structure due to cold water that advances fast from deeper parts of the lake along the shallow slope.

Using the time series of observed temperatures at the four thermistor chains, we can reconstruct a schematic diagram that describes how the thermocline interacts with the lake bed (Figure 6). During the downwelling phase of the internal seiche there is a small slope to the isotherms, so that they are slightly raised at inshore sites (Figure 6a). The stratification at the boundary is stronger than offshore stratification in the thermocline, and there is a very gradual temporal transition between cold and warm water temperatures at a fixed point. This conceptual picture is consistent with the previous descriptions of the benthic temperature structure during downwelling of the thermocline [14], [19]. In contrast, during the upwelling phase, the isotherms are tilted almost vertically where the thermocline intersects the lake bed. In addition, offshore temperatures are higher at a given depth than onshore leading to a tilt of the isotherms in the near-shore thermocline.

Based on the time of occurrence and distance traveled we estimate velocities of the front of up to 0.15 m s^−1^ between T3 and T1. This fast cold water front (bore) is similar to the internal “surge” that occurred in shallow water off the Scripps pier in La Jolla, California [41]. Similar observations of the shoaling of internal tidal waves in shallow coastal waters [42] have also found that the upwelling of colder water often results in the formation of a cold internal tidal bore that propagated at speeds of around 0.15 m s^−1^. These observations were made at a depth of 20 m and showed a very similar asymmetry in stratification between up- and downwelling phases, to the present observations.

Non-linear bores are a hydraulic jump and will propagate at a speed which is limited by the internal wave speed. Such a bore represents a flow regime transition from sub- to supercritical flows and therefore will have a Froude number of unity [43]. The mean speed *U_d_* can be calculated by
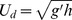
(6)with *h* being the thickness of the bore, and *g′* being the reduced gravity across the sharp interface (defined as *g′* = *(ρ_2_–ρ_1_*)/*ρ*_o_ ). We note that this speed is essentially the same as that at which a cold gravity current would intrude into a warm ambient, as experimentally described by [44] or [45]. The propagation speed of the cold water bore observed in Figure 5b–d can then be estimated from the temperature difference across the thermocline and the thickness over which the temperature inversions occur. For instance, during upwelling the temperature step at the sites T1–T3 (Figures 3e to 3f) is about *δT = *24°C −14°C = 10°C, so that *g*′ = 0.019 m s^−2^. A reasonable estimate of the height is *h = *1.4 m as observed as the thickness of the layer over which temperature inversions occur (Figure 4). This yields an average speed of *U_d_* = 0.16 m s^−1^ for upslope flow of cold water which agrees well with the estimate obtained from the onset of inversions at adjacent thermistor chains. Other internal bores can be observed during the other strong upwellings of the thermocline, such as around DOY 235 and 242. Not all of these events propagate all the way from T3 to T1 as the vertical movements of the thermocline were smaller in these cases. For those events the average speed is slightly less at 0.10 m s^−1^.

### Estimated Turbulence Near the Lake Bed

**Figure 7:**
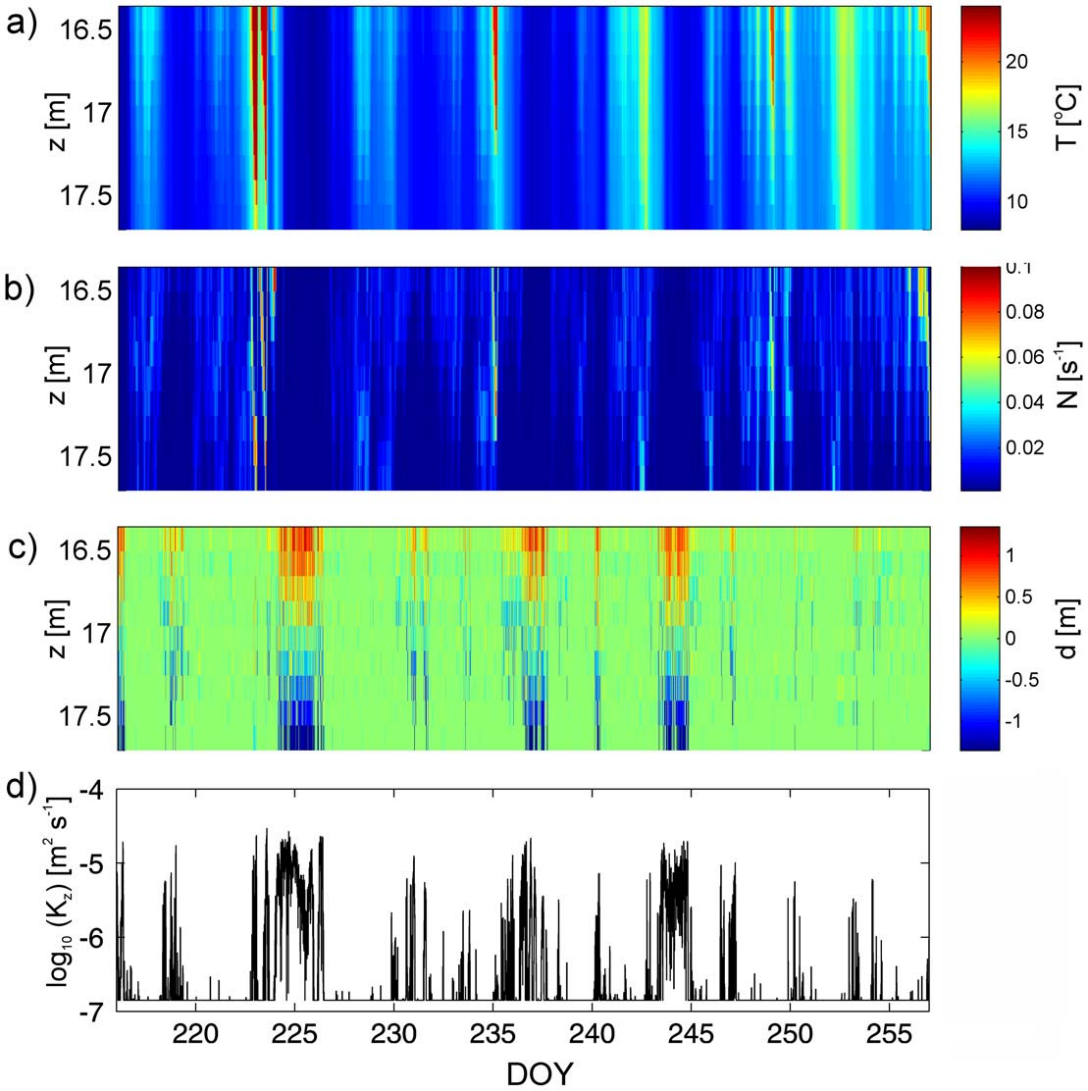
Near bed temperature time series and estimates of turbulent parameters at the benthic thermistor chain site T3. a) Observed temperature data. b) Buoyancy frequency *N* after reordering the temperature profiles to stable profiles at every time step. c) Estimated overturning displacements. d) Time series of the distribution of estimated eddy diffusivity averaged over the entire height of the benthic thermistor chain.

The time variation of the vertical eddy diffusivity can be estimated from the observed temperature inversions at the benthic thermistors. Similar turbulent mixing events were observed at all benthic thermistor chains but for simplicity we only show data for the T3 mooring. Figure 7 compares the observed thermal stratification (Figure 7a), the buoyancy frequency (Figure 7b) and derived overturning scales (Figure 7c) along with the estimated values of *K_z_* (Figure 7d) at T3. There is a significant correlation between the upwelling phases of the internal seiche and strong increases in *K_z_*. This means that most of the strong mixing events at the boundary of the lake are associated with the upward movements of the thermocline past the measurement location, for instance at DOY 225, 335 and 242. During upwelling these overturns can reach up to 1 m in vertical scale and consequently increase values of *K_z_* throughout the BBL. The mean diffusivity at T3 is *K_z_* = 1.48×10^−6^ m^2^ s^−1^ and maximum values reach *K_z_* = 1×10^−4^ m^2^ s^−1^. Generally, these values of diffusivities are consistent with vertical diffusivities near boundaries found by many observations in oceans and lakes (e.g. [1], [2], [46], [47]).

**Figure 8:**
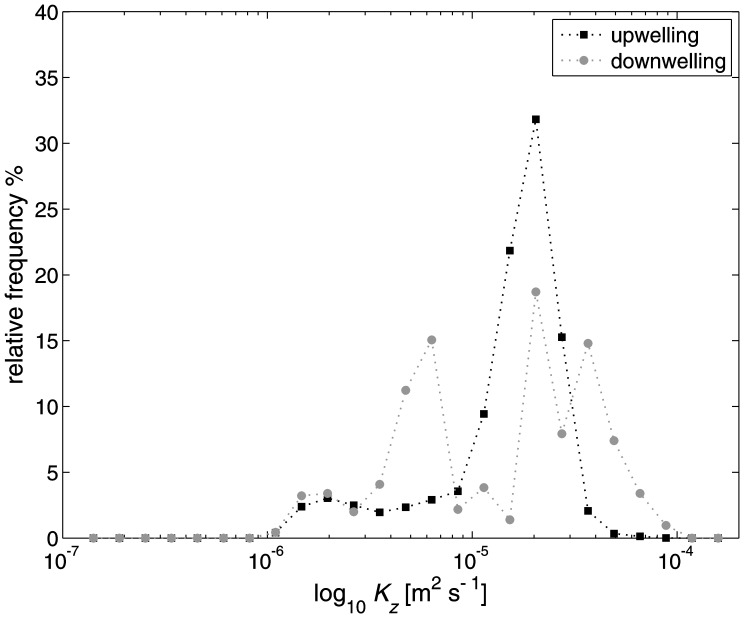
Frequency distributions of turbulent diffusivities at mooring T3. Estimated *K_z_* values during upwelling (black) and downwelling (gray) events. The significant asymmetry can be attributed to the unstable BBL stratification observed by temperature inversions during upwelling events at the lakebed.

There is a strong asymmetry in the frequency distribution of estimated turbulent diffusivities *K_z_* averaged during the up- and downwelling phase of the internal seiche (Figure 8). The up- and downwelling phases were identified by monitoring the bottom thermistor at T3. Upwelling of cold water is characterized by a period of decreasing temperatures. The time between a cooling event and when the temperature started to rise and exceed a certain threshold (12°C) was identified as an upwelling phase. On the other hand, downwelling of warm water was identified by the time when the temperature was above this threshold (12°C). The mean values of *K_z_* during upwelling are at the order of *O*(10^−6^) m^2^ s^−1^ but show a significant frequency peak around *K_z = _*2×10^−5^ m^2^ s^−1^ (Figure 8). During downwelling phases the mean values are generally one order of magnitude smaller with mean values of *K_z = _*2.35×10^−7^ m^2^ s^−1^ but can also reach values at the order of *O*(10^−4^). However, at the site T1 we experience much more rapid cooling associated with steep temperature gradients which lead to more turbulence and significantly larger values of *K_z_* during up- than during downwelling. Our findings are concurrent with observations by [4] who reported similar frequency distributions of estimated turbulent diffusivities *K_z_* for unstable upwelling and stably stratified downwelling BBLs due to shear-induced convection in Lake Constance. However, their mean values of *K_z_* = 5×10^−4^ m^2^ s^−1^ for down- and *K_z_* = 2×10^−3^ m^2^ s^−1^ for upwelling are one to two orders of magnitude larger. This might be attributed to several reasons, such as the reported large internal seiche amplitudes (up to 37 m) and the presence of weak stratification at the observation location at the depth of 100 m. In addition, local parameters such as the slope angle and roughness of the bed can influence the magnitude of turbulence in the BBL for both breaking NLIW and shear-induced convection [18], [30], [48]. Finally, the method used to estimate diffusivities might cause differences between the present observations and those in Lake Constance. For instance, using high-resolution velocity measurements [49] calculated diffusivities that were at the same order of magnitude as in this study, but one order of magnitude smaller than estimated in [4] who used a log-law approach. Nonetheless, all studies confirm that stable stratification during downwelling inhibits overturning and mixing in the water column whereas unstable stratification leads to convectively driven mixing processes during upslope flow [4]. We note that shear-induced convection in lakes seems to be different to observations in ocean basins where the Ekman transport in downwelling events resulted in more destabilizing effects and was the major contributor to boundary mixing [12].

Our findings suggest that shear-induced convection can be an important and episodic mixing process at depths where a thermocline intersects the sloping lake boundaries. This is particularly so, as we saw no evidence to suggest that breaking of NLIWs was an important process at this location in the lake. If the observed mixing within the wash zone of the thermocline can be projected onto other areas of similar depth and slope, such as the mostly shallow and spacious eastern basin of Lake Simcoe, it would greatly increase the area subjected to intermittent turbulent fluxes. Our estimates of *K_z_* values during upwelling could then be valid for more than 20% of the area of the lakebed. Extensive thermocline movements and related mixing processes are often characterized by *L_N_* [1], [30]. Our results in Figure 4 suggest that enhanced boundary mixing occurs in Lake Simcoe following large upwelling of the thermocline due to large amplitude seiches. These, generally occurred after periods when *L_N_* <2 in Lake Simcoe. However, we acknowledge that in a large lake Coriolis effects could also be important, and these are not included in a simple dimensionless parameter such as the *L_N_*.

### Conclusions

Internal seiches were greatest after storm events, when the thermocline showed significant vertical excursions. This resulted in a large lateral movement of the position where the thermocline intersected the shallow sloping lakebed of Lake Simcoe. During upwelling phases of the internal seiche, a sharp front formed on the upwelling thermocline, so that the upwelling cold waters resembled an internal bore which propagated up the gentle slope with speeds between 0.05 to 0.15 m s^−1^. During the passage of the bore there were strong temperature inversions that lead to enhanced mixing in the BBL. In contrast during periods of downwelling the BBL revealed a stable stratification which was as high as 10°C m^−1^ and a strong reduction in turbulent diffusivities. Our main result is that turbulent diffusivities in the BBL will only be intermittently high, following large excursions of the thermocline in response to strong wind events.

We did not see any evidence to suggest that the breaking of non-linear internal waves was a significant source of turbulence at the location of the thermistor chains in Lake Simcoe, where it appears that shear-driven convection is a more important process for setting benthic turbulence levels. However further study is required to determine the exact conditions when either process of NLIW breaking or shear induced convection would be predicted to be dominant in any given lake.
